# Brain Activity Related to Sound Symbolism: Cross-modal Effect of an Aurally Presented Phoneme on Judgment of Size

**DOI:** 10.1038/s41598-019-43457-3

**Published:** 2019-05-07

**Authors:** Sachi Itagaki, Shota Murai, Kohta I. Kobayasi

**Affiliations:** Graduate School of Life and Medical Sciences, Doshisha University, 1-3 Tatara Miyakodani, Kyotanabe, 610-0394 Japan

**Keywords:** Language, Human behaviour

## Abstract

Sound symbolism is the idea that a sound makes a certain impression (e.g., phoneme “p” is associated with an impression of smallness) and could be the psychological basis of the word–meaning association. In this study, we investigated the neural basis of sound symbolism. Subjects were required to compare the visual sizes of standard and target stimuli while listening to syllables assumed to create either a larger or smaller impression. Stimulus–response congruence is defined as the agreement between the target size and the syllable’s impression. Behavioral data showed that the subjects displayed a longer reaction time under the incongruent condition than under the congruent condition, indicating that they tended to associate the object size with certain syllables. We used functional magnetic resonance imaging to evaluate the cerebral activity during the task, and found that both semantic- and phonetic-process-related areas of the brain (left middle temporal gyrus and right superior temporal gyrus, respectively) were activated under the incongruent condition. These results suggest that these regions are associated with the incongruence of sound symbolism.

## Introduction

It has been assumed that in natural language, there is no particular regularity in the correspondence between an object and the acoustic properties of the word that describes that object^1^. However, a phenomenon called “sound symbolism” or “phonetic symbolism” in the association between meanings and sounds (i.e., phonemes) has been confirmed by several studies^2–11^. The idea underlying this phenomenon is that a sound itself makes a certain impression, which then serves as the psychological basis for the word–meaning association.

The most famous example of this phenomenon is the so-called “bouba/kiki effect”^12^, which involves asking a participant to name spiky and round shapes using only the sounds “bouba” and “kiki”. According to the results, most people associate “bouba” with “round” and “kiki” with “spiky”. Moreover, this effect has been observed regardless of the subject’s age or native language. Most sound symbolism studies have used a relatively naturalistic approach^9–11,13^. In one study, in an attempt to illustrate the effect of each phoneme, subjects were asked about their impressions of an artificial word^11^. These studies successfully detected a relationship between the acoustic features of a stimulus word and the impression left by or the response to these features. However, the neural basis of sound symbolism is less well understood.

Kovic and colleagues analyzed the neural processes underlying novel word–visual object matching using electroencephalography (EEG)^14^. Their results showed that sound–symbol matching evoked an early negative EEG response, indicating that such matching involved early sensory processes. Recent EEG research with an 11-month-old infant showed that large-scale synchronization within the left hemisphere was sensitive to the sound–symbol correspondence^15^. These results suggest that the preverbal infant is capable of mapping an auditory stimulus onto a visual experience by recruiting a multimodal perceptual processing system. One early neuroimaging study of sound symbolism (Japanese onomatopoeia) was conducted by Osaka and colleagues, who used functional magnetic resonance imaging (fMRI) to investigate how onomatopoeic words modified the activity of different cerebral regions^16,17^. Recently, Kanero *et al*. studied the neural basis of sound symbolism with fMRI using the written text of Japanese mimetic words and moving images^18^. Their results showed that the right temporal region was involved in processing Japanese mimetic words. Here, we used the sounds of phonemes as auditory stimuli, and examined how different aspects of sound symbolism (phoneme vs mimetic word) and different modalities of stimulus presentation (visual vs auditory) affect the activities of the right temporal and other brain regions.

**Figure 1:**
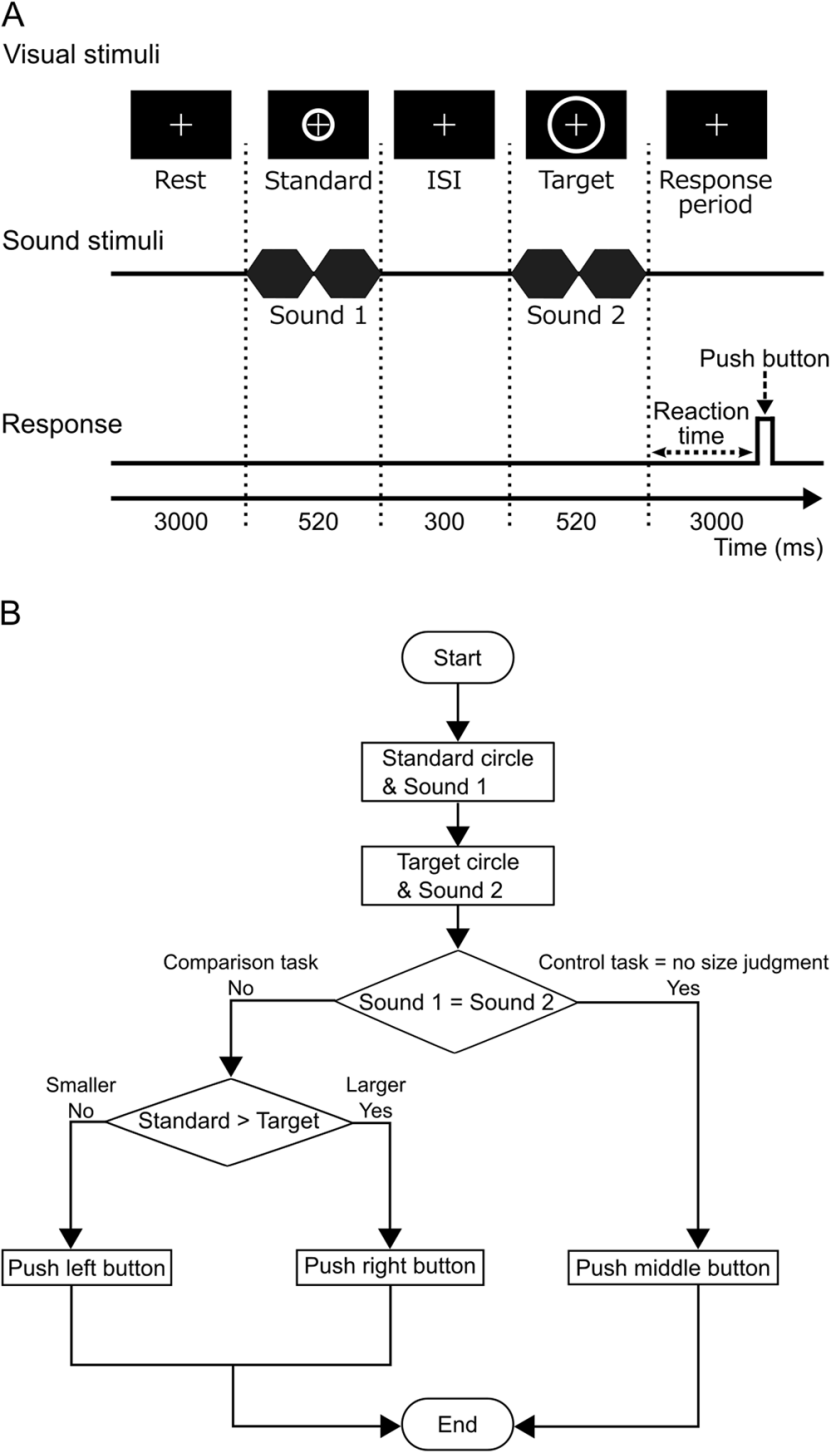
(**A**) Illustration of the experimental design. Each trial started with a rest period (3,000 ms). The standard stimulus (520 ms) was then presented, followed by the target stimulus (520 ms) after a 300 ms interstimulus interval (ISI). Subjects were instructed to respond to the task during the response period (3,000 ms) via a button device, using the fingers of their left hand. A red cross was always presented as the fixation point. (**B**) Flow chart illustrating the experimental process. Subjects were instructed to push the middle button to respond to the control task and to push the left or right button to respond to the comparison task. Button assignment (which button represented a specific answer) in the comparison task was changed between subjects.

In this study, we examined the effect of a phoneme (the basic unit of sound within a word) on the judgment of the size of a visual stimulus and the related brain activity. Subjects were required to perform two types of task, a comparison task and a control task (Fig. 1). In the comparison task, they compared the visual sizes of standard and target stimuli. The target stimulus was either smaller or larger than the standard by ±5%, ±10%, or ±20% of its diameter, and was displayed to the subjects while they were listening to the syllable “bobo” or “pipi”. Previous research into sound symbolism predicted a tendency to pair the former sound with a larger object and the latter with a smaller object^13^. The control task was designed to prevent subjects ignoring the sound stimuli.

## Results

We analyzed the reaction times (RTs) for the compared tasks. Several studies have shown that the phonemes “b”, “d”, “g”, and “o” elicit “larger” reactions, whereas “p”, “t”, “k”, and “i” elicit “smaller” reactions^13,19^. Therefore we assumed that “bobo” and “pipi” would induce “larger” and “smaller” impressions, respectively. We defined the congruent and incongruent conditions as follows and analyzed the data accordingly. Under the congruent condition, the target visual stimulus was consistent with the reaction to the sound (i.e., the larger target was presented with “bobo” or the smaller with “pipi”). Under the incongruent condition, the target visual stimulus was inconsistent with the reaction to the sound (i.e., the larger target was presented with “pipi” or the smaller one with “bobo”).

**Figure 2:**
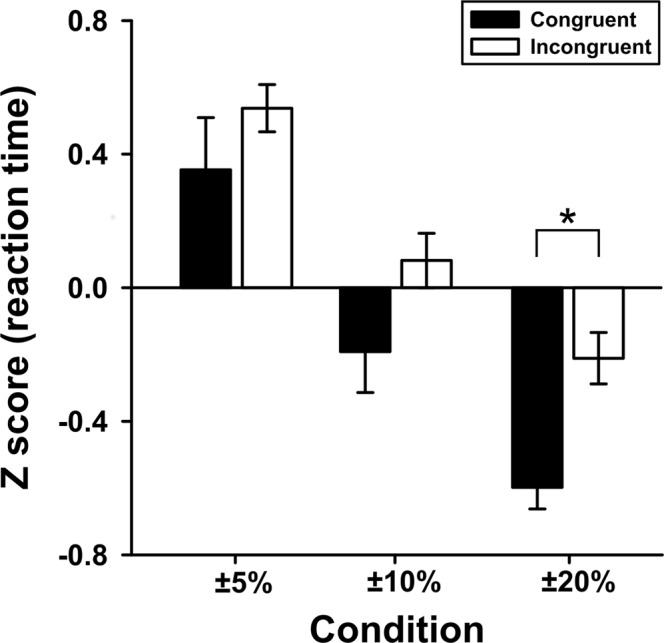
Average reaction time under each circle-size condition (Z-score). The vertical axis represents the Z-score and the horizontal axis represents the target size. Error bars represent the standard errors of the means. The Z-score was greater under the incongruent condition than under the congruent condition for all target-size conditions; the difference under the ±20% target-size condition was significant (t = −5.93, *p* < 0.001).

Eleven subjects performed well on the comparison task. The mean correct response rate was 94.1%. The differences in the RTs under the congruent and incongruent conditions are shown in Fig. 2. The mean RT decreased as the size difference between the targets increased from ±5% to ±20%. The mean RT under the incongruent condition was longer than that under the congruent condition for all target sizes. However, this difference was statistically significant only when the target was ±20% of the standard (t = −5.93, *p* < 0.001, *t* test with Bonferroni correction).

**Figure 3:**
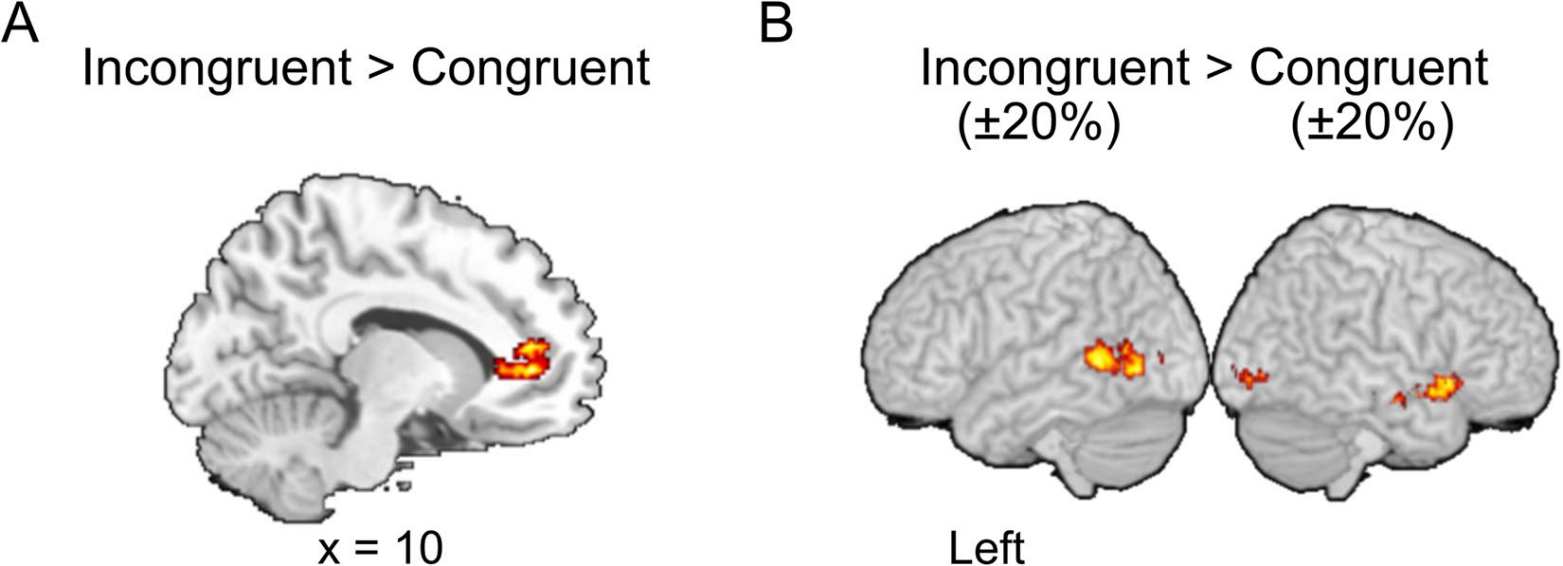
(**A**) Activation maps under the incongruent condition. The brain region showing greater activity under the incongruent condition than under the congruent condition is the ACC (*p* < 0.001 with a cluster-level family-wise error ([FWE] correction of *p* < 0.05). (**B**) Activation maps under ±20% incongruent conditions. Shown are the brain regions with greater activation during the ±20% incongruent condition than during the ±20% congruent condition (*p* < 0.005 with a cluster-level FWE correction of *p* < 0.05). Significant regions were superimposed on a standard brain template from MRIcro software.

In this study, we investigated the brain region associated with sound symbolism by contrasting the incongruent condition with the congruent. The bilateral anterior cingulate cortex (ACC) was activated under the incongruent minus congruent condition for all target sizes (Fig. 3A, Table 1).

**Table 1:** MRI activation clusters derived from comparison of the incongruent and congruent conditions.

Brain region	MNI coordinates			*Z*-score	Cluster size (voxels)
	x	y	z		
Right anterior cingulate cortex	**10**	42	2	4.91	569
Left anterior cingulate cortex	**0**	32	8	4.78	

Note: Coordinates are in MNI (Montreal Neurological Institute) space. Included are clusters that survived the threshold at a voxel level of *p* < 0.001 with a cluster-level family-wise error (FWE) correction of *p* < 0.05.

**Figure 4:**
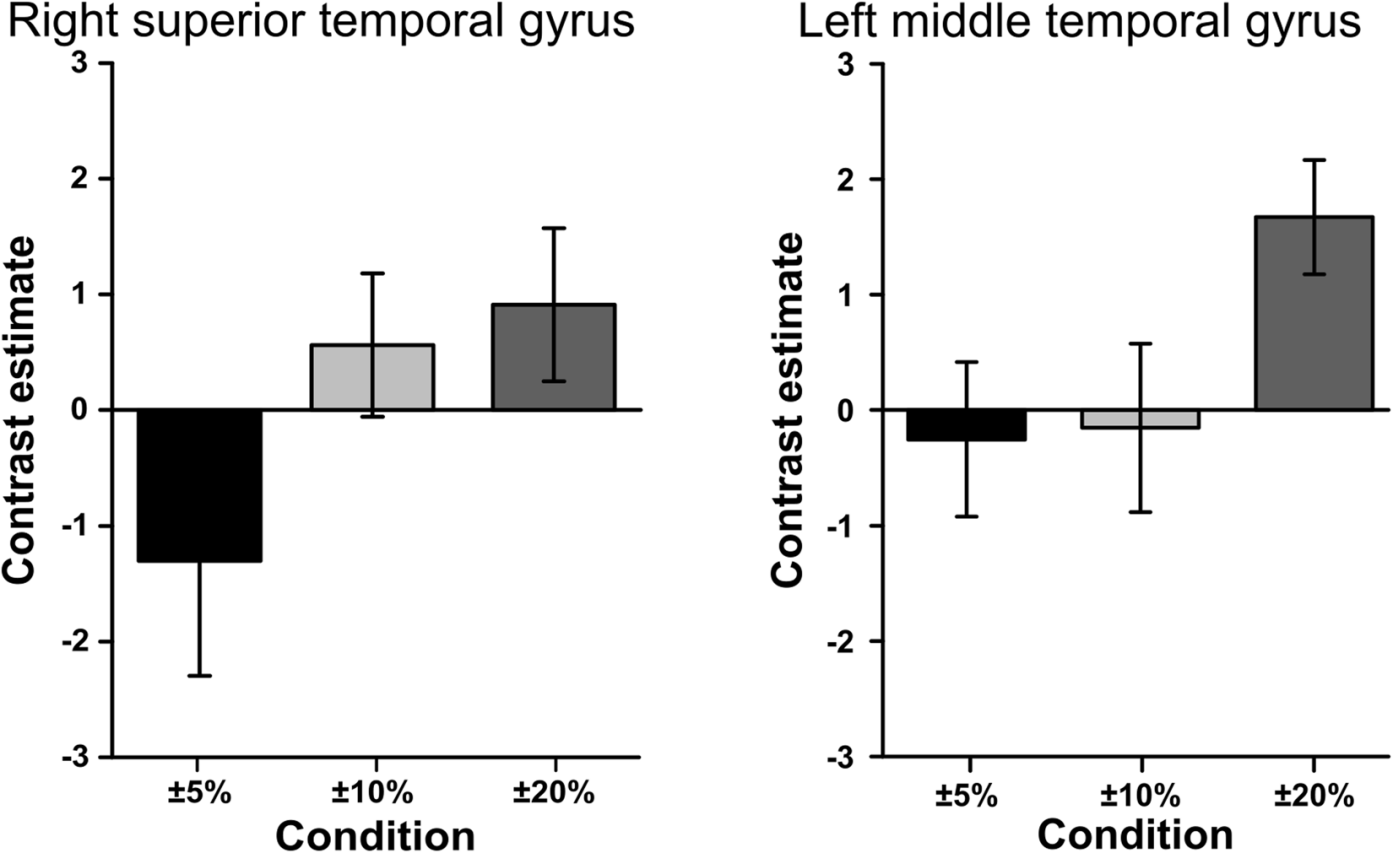
Contrast estimate for each target size, calculated by subtracting the congruent from the incongruent condition, in the right superior temporal gyrus and left middle temporal gyrus. The ROIs were spheres with a radius of 4 mm. Error bars represent standard errors of the means. The contrast estimate was greater for a difference of ±20% than for a difference of ±5% or ±10% in each region.

The activation of the right lingual gyrus, left middle temporal gyrus (MTG), and right superior temporal gyrus (STG) was observed under the ±20% incongruent minus ±20% congruent condition (Fig. 3B, Table 2). The contrast (incongruent − congruent) for each target size condition in each of these brain regions is presented in Fig. 4. There was no significant activation under the ±20% congruent minus ±20% incongruent condition.

**Table 2:** MRI activation clusters derived from the comparison of the ±20% incongruent condition and the ±20% congruent condition.

Brain region	MNI coordinates			*Z*-score	Cluster size (voxels)
	x	y	z		
Right lingual gyrus	**32**	**−60**	**−2**	4.35	774
Right inferior occipital gyrus	**40**	**−60**	**−2**	4.14	
Right lingual gyrus	**18**	**−40**	**−2**	3.95	
Right inferior occipital gyrus	**36**	**−62**	**0**	3.89	
Right calcarine cortex	**32**	**−64**	**6**	3.88	
Left middle temporal gyrus	**−48**	**−44**	**0**	4.22	1040
Left middle occipital gyrus	**−30**	**−76**	**8**	4.06	
Left middle temporal gyrus	**−48**	**−50**	**−2**	4.05	
Left middle temporal gyrus	**−44**	**−50**	**0**	4.01	
Left lingual gyrus	**−26**	**−54**	**−4**	3.76	
Right frontal operculum	**50**	**16**	**−6**	4.00	551
Right temporal pole	**44**	**10**	**−14**	3.73	
Right posterior orbital gyrus	**28**	**26**	**−10**	3.73	
Right planum polare	**44**	**−6**	**−10**	3.71	
Right superior temporal gyrus	**48**	**−10**	**−12**	3.57	

Note: Coordinates are in MNI space. Included are clusters that survived the threshold at a voxel level of *p* < 0.005 with a cluster-level FWE correction of *p* < 0.05.

The contrast estimates (incongruent − congruent) for the right STG and left MTG under each condition were evaluated with a region-of-interest (ROI) analysis with leave-one-subject-out cross-validation, and are shown in Fig. 4, in which a positive value indicates stronger activation during the incongruent condition than during the congruent condition. The difference at ±20% was more prominent than that at ±5% or ±10% in each region.

## Discussion

The RT under the congruent condition was shorter than that under the incongruent condition, suggesting that the effect of sound symbolism was observed under our experimental setting (the phoneme as a sound stimulus). However, the efficacy of sound symbolism differed with the target size, and only the ±20% condition yielded a significant difference between the congruent and incongruent conditions. Because the RTs were more strongly affected by the target size than by the congruent–incongruent difference (Fig. 2), it is not surprising that the effect of sound symbolism was minimal under some target conditions.

The bilateral ACCs were more strongly activated under the incongruent condition than under the congruent condition. The activation of the dorsal ACC has been associated with cognitively demanding tasks, typically involving response conflict, such as the Stroop task^20,21^. In this study, we observed a more pronounced BOLD response in the dorsal ACC, suggesting that the task performed under the incongruent condition was more cognitively demanding for the subjects. Together with the longer RTs, the activation of the dorsal region of the ACC may reflect the Stroop-like interference between the phoneme and the visual magnitude of the stimulus.

The left MTG was more strongly activated under the ±20% incongruent condition than under the ±20% congruent condition. The results of the ROI analysis of the peak region are plotted in Fig. 4. The left MTG has been identified in previous studies as a brain region related to semantic association (see review by Price)^22^. This region is also very similar to the area that was activated under the incongruent condition in a priming study using EEG^23^, in which congruence was based on the relationship between a picture and an environmental sound (i.e., an animal and its vocalization). That study demonstrated that the left MTG is involved in cross-modal semantic-matching processes. Our results imply that sound symbolically matching between the target size and a phoneme is also processed in the MTG.

The right STG was more strongly activated under the ±20% incongruent condition than under the ±20% congruent condition. The ROI analysis of the peak region (Table 2) revealed that the activation of the STG (incongruent minus congruent) was more prominent as the size difference between the targets increased from ±5% to ±20% (Fig. 4), indicating that the efficacy of the sound symbolism correlated with the activation of the region. Many previous studies have identified the STG as a primary region for speech perception^22^. Interestingly, activities in the right STG have been associated with the incongruence between emotional prosodic cues and other information (speech content^24^ and facial expression)^25^. Our results suggest that the right STG is part of a brain network involved in processing conflict in phonemic sound symbolism in addition to emotional prosodic information.

Kanero and colleagues (2014) reported that the right posterior STG was more strongly activated when subjects were evaluating matched pairs of mimetic words and moving images than when they were evaluating mismatched pairs. However, our analysis detected no significant activation in this region. Although the functional localization of the right STG is still contentious, several researchers have reported that the anterior region is strongly associated with phoneme perception^26,27^. The stimulus difference (mimetic word in Kanero’s study or phoneme in our study) could contribute to the difference in the activated area. In addition to analyzing the stimulus difference, they asked their subjects to actively report their impression of mimetic words, whereas our subjects were engaged in a visual discrimination task, and were not asked to report their impression of the phoneme. The observed differences in activation area could be attributable to these task differences. Further research is required to investigate the sound symbolic effect on both phonemes and mimetic words within the same experimental paradigm to detect the functional segregation, if any, of the right STG into the different aspects of sound symbolism. There are several limitations of this study. The number of subjects is relatively small, and only two types of speech sounds were tested; therefore, our data should be treated as a pilot study to draw general conclusion on the neural processing of sound symbolism. We still believe that our data indicate the involvement of bilateral temporal regions (i.e., the right STG and left MTG) in the sound-meaning association.

## Materials and Methods

### Subjects

Fourteen subjects (four females and ten males; aged 21–26 years) participated in the fMRI experiment after they had provided their written informed consent. All the subjects were right-handed native Japanese speakers. None of the participants had any knowledge of sound symbolism or the experiment. The data from three participants were excluded because of artifact or inadequate task performance (e.g., head movement >3 mm). The experimental protocol was approved by the Research Ethics Committee on human subjects of Doshisha University, and the study carried out in accordance with the guidelines of the committee.

### Experimental apparatus

Each subject was positioned supine in an MRI scanner. The sound stimuli were presented through MRI-compatible headphones (Kiyohara Optics Inc., Tokyo, Japan), and the visual stimuli were presented with a projector and mirror system. The subjects viewed the visual stimuli projected onto a mirror placed 18 cm in front of their eyes, and they pushed buttons (Current Designs, Inc., PA, USA) with their left hand to respond. Experiment control software (Presentation®; Neurobehavioral Systems, Inc., Albany, CA, USA) was used to synchronize the experimental procedures with the fMRI scans.

### Visual stimuli

In this experiment, we examined the effect of sound symbolism when judging the size of a visual stimulus. The visual stimulus, a gray circle that looked like a doughnut, was presented on a frosted screen at the end of the scanner bore, and could be seen by the subject via a mirror mounted on the head coil. The standard stimulus had an outer circle of 300 pixels and an inner circle of 280 pixels. The target stimulus was either smaller or larger than the standard stimulus by ±5%, ±10%, or ±20% of its diameter. In total, seven sizes (one for the standard and six for the targets) were used. Each stimulus was presented twice for 200 ms, with an interstimulus interval (ISI) of 120 ms (Fig. 1A). A red cross (34 pixels) was always presented as the fixation point at the center of the screen.

### Sound stimuli

The sound stimuli were “bobo” and “pipi”. A publicly available sound dataset (FW03; NTT Communication Science Laboratories, Kanagawa, Japan) was used to create the sounds. All sounds were recorded at a sampling frequency of 48 kHz and a quantization of 16 bits. The single-syllable utterances “bo” and “pi” were spoken by a male, and these were duplicated to produce the sound stimuli “bobo” and “pipi”, respectively. These sound stimuli have no accent, therefore they were not similar to any word. The duration of the sound was 520 ms, and the stimulus amplitude was 64 dB sound pressure level (SPL). According to previous research, the sound “pi” was louder than “bo” by about 1.4 dB in subjective loudness^28^. The sound stimulus was synchronized with the visual stimulus. In an additional experiment, the participants were asked to evaluate the size of each sound stimulus by selecting labeled pictures of five Russian nested dolls of different sizes (doll 5 was the largest)^8,11^. The mean scores for “bobo” and “pipi” were 4.0 and 2.3, respectively, and no participant ever scored “bobo” smaller than “pipi”. Therefore, we confirmed that “bobo” created a larger impression than “pipi”.

### fMRI parameters

Functional images of brain activity were acquired with a 1.5-T MRI system (Echelon Vega, Hitachi Medical Corporation, Tokyo, Japan) as T2*-weighted images using a gradient echo–echo planar imaging (GE–EPI) sequence with a resolution of 3 × 3 × 5 mm voxels (30 axial slices; field of view [FOV]: 192 mm; matrix: 64 × 64; repetition time [TR]: 3000 ms; echo time [TE]: 50 ms; flip angle [FA]: 90°). The first five scans were discarded to avoid magnetic saturation effects. A structural T1 image was acquired at a resolution of 1 × 1 × 1 mm using a three-dimensional gradient echo inversion recovery (3D-GEIR) sequence (192 slices, 1 mm thick; sagittal; FOV: 256 mm; matrix: 256 × 256; TR: 9.7 ms; TE: 4 ms; T1: 1045 ms; FA: 8°).

### Procedure

The subjects were asked to judge the difference in the sizes of the standard and target stimuli^29^. Each trial began with a 3,000 ms rest period. The standard stimulus was then presented for 520 ms, followed by a 300 ms ISI. The target stimulus was then presented for 520 ms, followed by a response period of 3,000 ms (Fig. 1A). The screen was black during the rest and response periods. After the subject had responded to the task by pressing a button, the next trial began automatically. When the sound (sound 2) presented with the target stimulus was identical to the sound (sound 1) presented with the standard stimulus, the subject had to press the middle button, regardless of the visual stimulus (control task). When the sound stimulus presented with the target stimulus differed from that presented with the standard stimulus, the subject had to respond according to whether the target circle was smaller or larger than the standard (Fig. 1B). The subjects used their index and ring fingers to press the left and right buttons, respectively (comparison task). The button assignment (i.e., which button represented which answer) was changed between subjects. There were six combinations of visual stimuli (one standard × six targets) and four combinations of sound stimuli (two sound 1 × two sound 2), yielding a total of 24 stimulus combinations. The entire stimulus set was randomized to create one block (24 trials), and each session consisted of three blocks. In the behavioral experiment, each subject completed one session (72 trials).

### Analysis

The functional imaging data were processed with the SPM12 software (Wellcome Department of Cognitive Neurology, London, UK). For realignment, the image was spatially normalized to an EPI template in Montreal Neurological Institute (MNI) space, and smoothed with an 8 mm full width at half maximum Gaussian kernel. To examine the brain activation associated with phonetic symbolism, we created different types of contrast images (congruent minus incongruent for all target sizes combined, incongruent minus congruent for all target sizes combined, congruent minus incongruent for ±20% target sizes, and incongruent minus congruent for ±20% target sizes). These effects were modeled as stick functions convoluted with the canonical hemodynamic response function. The movement parameters of the realignment corrections were included in the model as covariates of no interest. Activated areas were depicted on a standard human brain template from MRIcro software (version 1.40, www.mccauslandcenter.sc.edu/crnl/mricro).

To further clarify the effect size, we used a ROI analysis of the contrast estimates in the right STG and left MTG. Each ROI was constructed with leave-one-subject-out cross-validation to avoid any bias arising from nonindependence^30^. Briefly, the data for each subject were iteratively left out of the group. The resulting group (N − 1 subjects) analyses returned peak coordinates of the ROIs (spheres with a 4 mm radius) for the subject who was left out, and the average responses in the ROIs in the right STG and left MTG were estimated.
